# Stacked scattering: The key to bright flowers lies in the mesophyll

**DOI:** 10.1002/ajb2.70104

**Published:** 2025-09-25

**Authors:** Larissa De Paola, Thomas A. Veldhuis, Marjan Kraaij, Doekele G. Stavenga, Kira J. Tiedge, Casper J. van der Kooi

**Affiliations:** ^1^ Groningen Institute for Evolutionary Life Sciences University of Groningen Groningen The Netherlands

**Keywords:** floral anatomy, floral epidermis, flower color, *Hypericum*, mesophyll, *Oenothera glazioviana*, scattering coefficient, spectroscopy, *Tropaeolum majus*

## Abstract

**Premise:**

The coloration of flowers is caused by wavelength‐selective absorption by pigments and scattering of light by floral structures. Although the molecular, physiological, and chemical properties of floral pigments have been studied in considerable detail, how floral structures contribute to the visual signal remains largely unknown. A flower can be considered as a stack of layers, where each layer is characterized by specific pigmentation and scattering properties. Quantifying the contribution of different floral layers to visual signalling aids our understanding of the origin and maintenance of Earth's resplendent flora.

**Methods:**

We quantified the contribution of the cuticle, epidermal cell layer, and the mesophyll to the reflection of light by flowers for nasturtium (*Tropaeolum majus*), a St. John's wort hybrid (*Hypericum* ‘Hidcote’), and the large‐flowered evening primrose (*Oenothera glazioviana*). The obtained experimental and modelling data allowed the quantification of the absorption and scattering of light by different floral layers.

**Results:**

The mesophyll was by far the strongest reflecting layer. The reflectance from the epidermal layer was minor, and the cuticle reflected a very small percentage of the total. The strong scattering by the mesophyll is caused by its inhomogeneity and thickness.

**Conclusions:**

The strong light‐scattering by the mesophyll crucially determines a flower's visual signal, whereas the cuticle and epidermal cells contribute less than generally assumed. Mesophyll thickness, cell properties and inhomogeneity, including porosity, are essential components of the flower's optical toolkit.

Floral visual signals aid plants in attracting pollinators (Chittka and Menzel, 1992; Lee, 2007; Schiestl and Johnson, 2013; van der Kooi et al., 2019). The large diversity of pollinator species and pollination strategies have given rise to a tremendous variety of floral colors. Flower coloration is based on two fundamentally different optical principles: (1) wavelength‐selective absorption of light by floral pigments and (2) scattering of incident light by floral structures (van der Kooi et al., 2016). The modulation of the visual signal by floral pigments is well studied (Chittka and Menzel, 1992; Mol et al., 1998; Yoshida et al., 2006; Rausher, 2008; Dyer et al., 2012; Wessinger and Rausher, 2012; Sheehan et al., 2016; Narbona et al., 2021; Sapir et al., 2021), but the contribution of the various floral structures to the scattering of light is poorly understood.

Optically, the signalling structures of the perianth (e.g., petals, sepals, tepals, and ligules) can be considered as a stack of layers, where each layer has specific reflection and absorption characteristics (Figure 1). The first layer is the cuticle, a thin waxy substance that covers the outer surface of the epidermal cells. The epidermis comprises one cell layer of quasi‐ordered cells (Figure 1, E). Below the epidermis lies the mesophyll, which comprises a thick layer of cells of varying sizes with intermittent air gaps. The shape and size of cells that constitute the epidermis and mesophyll vary among species (Kay et al., 1981; Whatley, 1984; van der Kooi et al., 2016; Schreel et al., 2024). Reflection of light occurs at the boundary of two media with different refractive indices, such as an air‐cell wall interface (Vogelmann, 1986). In other words, a structure's inhomogeneity (the number of boundaries per unit thickness) determines its reflectance. For flowers, some of the incident light is reflected by the surface (*R*_s_ in Figure 1), though most light enters the flower where irregularly shaped cellular structures diffusely scatter light (*R*_i_ in Figure 1). Interior, light‐scattering structures can be cell walls, vacuoles, and starch granules. The relative importance of different floral layers (e.g., the epidermis and mesophyll) for scattering light has been studied for only a few taxa and specific structures, such as glossy buttercup flowers (Parkin, 1935; Vignolini et al., 2012; van der Kooi et al., 2017), Baccara roses (Biran et al., 1974), the Chilean bellflower (Stavenga and van der Kooi, 2016) and snapdragon mutants (Gorton and Vogelmann, 1996).

**Figure 1:**
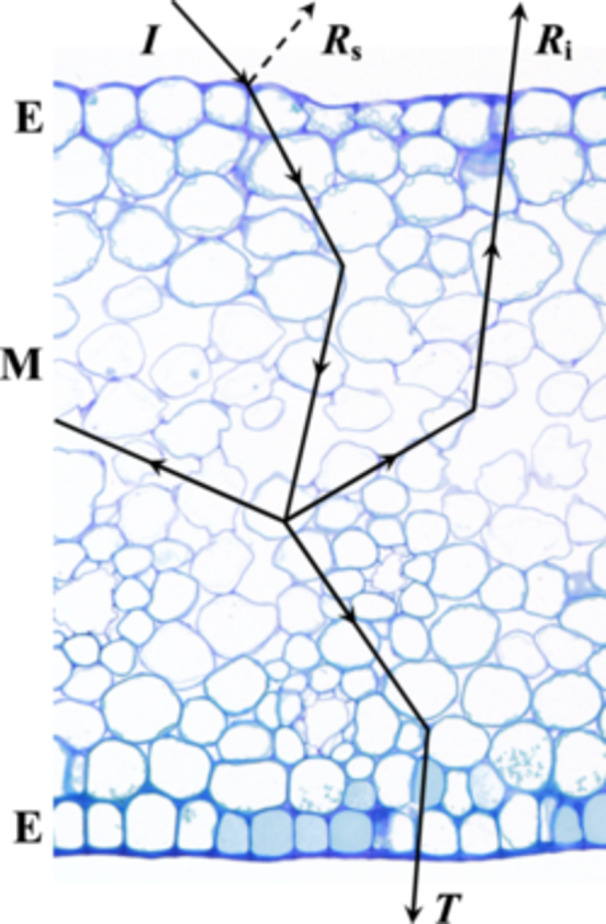
Schematic illustration of light propagation (black arrowed lines) in a petal (fixed petal of *Hypericum* ‘Hidcote’ stained with toluidine blue). The adaxial (top) and the abaxial (bottom) epidermis (E) are separated by the mesophyll (M). Part of the incident light (*I*) is reflected by the flower's outer surface (*R*_s_). The remaining light is scattered by cell walls, air gaps, and other internal structures or is absorbed by pigments. Part of the reflected light is backscattered from the interior (*R*_i_) or transmitted through the flower (*T*).

Understanding the tuning of floral visual signals to the visual systems of pollinators is a long‐standing question in pollination biology (Chittka and Menzel, 1992; Dyer et al., 2012; Schiestl and Johnson, 2013; van der Kooi et al., 2023). Furthermore, scattering of light by floral structures is important for abiotic factors, such as temperature regulation of the reproductive organs and internal reflection of (harmful) UV irradiance on pollen (Kevan, 1975; Koski and Ashman, 2015; González Moreno et al., 2022). To better understand the evolution of floral visual signals and plant–pollinator co‐evolution, it is important to know how different floral structures modulate the visual signal. Here, we studied the relative contributions of the floral layers to the visual signal in three flowering species: nasturtium (*Tropaeolum majus*, Tropaeolaceae), a St. John's wort hybrid (*Hypericum* × *hidcoteense* ‘Hidcote’, Hypericaceae) and the large‐flowered evening primrose (*Oenothera glazioviana*, Onagraceae). Spectrophotometry, anatomical observations, and calculations of the absorption and scattering coefficients illustrate how inhomogeneity and petal thickness can modulate the visual signal. Our results reveal that the mesophyll is a key contributor to floral light scattering and floral reflectance.

## MATERIALS AND METHODS

### Plant material and photography

Flowers of *Tropaeolum majus*, *Hypericum* ‘Hidcote’, an artificial hybrid between *H. calycinum* and *H*. × *cyathiflorum* (Maggi et al., 2008), and *Oenothera glazioviana* (Dietrich, 1997) were collected from different roadside locations on and near the Zernike Campus in Groningen, The Netherlands. These species were selected because they were readily available and their flowers have excellent resistance to mechanical damage and manipulation, allowing us to consistently and reliably dissect a petal with tweezers, one layer at a time. For each species, three flower samples were collected from different individuals. To visualize the appearance of the intact flowers, we photographed the samples using a digital camera (Nikon D70) equipped with an F Micro‐Nikkor macro lens (60 mm, f2.8, Nikon, Tokyo, Japan).

### Petal peeling, slide preparation, and spectrophotometry

To study the optical traits of the different floral layers, we used tweezers to gently peel off both epidermal layers and thus isolate the epidermal and mesophyll layers of a petal from each of the three flowers collected for each species. For each species, we measured absorbance and transmittance spectra (if possible) for an intact petal (i.e., no layers removed), isolated adaxial epidermis, isolated mesophyll, and a petal with the mesophyll exposed (i.e. without adaxial epidermis). However, the epidermal layers of *Hypericum* ‘Hidcote’ were too delicate to be removed intact, and the mesophyll layer of *T. majus* was too thin to be isolated from the epidermal layers. Each isolated layer was mounted on a custom‐made plastic black object slide (75 × 25 mm) with a central 4 mm aperture, which enabled the reflectance, transmittance or absorbance of the layer to be measured. All samples were measured immediately after preparation to minimize turgor loss and tissue damage.

The reflectance and transmittance spectra of isolated structures were measured using an integrating sphere (4 mm measurement diameter), which was connected to an Avaspec‐2048 CCD detector array spectrometer and illuminated using an Avalight‐DHS light source (Avantes, Apeldoorn, The Netherlands). An Avantes WS‐2 white diffuse reference tile was used as the white reference. For reflectance measurements, the sample was placed on the sphere's aperture and illuminated from inside the sphere. For transmittance measurements, the sample was placed on the aperture but illuminated from outside the sphere through a fiber (van der Kooi et al., 2016). The adaxial side of the sample always faced the detector. The transmittance of the various layers was measured with a microspectrophotometer (MSP) connected to the spectrometer mentioned above, which yields an estimate of the amount and type of floral pigment (Vukusic and Stavenga, 2009; Stavenga and van der Kooi, 2016). The measured area was a square with a side length of 10 µm. Sections of each isolated layer were then placed on microscope slides in immersion oil and observed with the MSP.

### Anatomy

To visualize the pigment content, we embedded small segments of fresh flowers in 6% w/v agarose and cut transverse sections (25 µm thick) using a sharp razor blade on a microtome (Kraaij and van der Kooi, 2020). Sections were photographed with an inverted Nikon Diaphot 300 microscope (Nikon, Amsterdam, Netherlands), coupled with a Nikon D3200 digital camera. To measure the layer thickness, we fixed small portions of petals in FAA_50_ (3.7% formaldehyde, 50% ethanol, 5% acetic acid v/v/v) for ~20 h at 4°C under a mild vacuum. Samples were dehydrated in an ethanol series (50%, 70%, 90%, 95%, absolute ethanol, each step for 1 h; absolute ethanol overnight). Samples were infiltrated under a mild vacuum with Technovit 7100 (Hereaus Kulzer, Germany) in 1:1 and 3:1 T7100–absolute ethanol, each step for 2 h, then 100% T7100 for 1 h and fresh 100% T7100 overnight. The resin was then polymerized according to the manufacturer's instructions. Prior to embedding, 0.6 mL of polyethylene glycol 400 was added to the embedding solution to soften the resin block for sectioning (Yeung and Chan, 2014). Samples were cut into sections 5 µm thick with 0.2 mm solid tungsten carbide blades (Sollex AB, Malmö, Sweden) on a rotary microtome (Beck, London, UK). Sections were stained with aqueous toluidine blue (0.05% w/v) and Sudan IV (0.1% w/v in isopropanol) to highlight the internal anatomy and cuticle, respectively. The dimensions of each structure (adaxial epidermis, abaxial epidermis, mesophyll, and the intact petal) were observed using ImageJ (v1.8.0, National Institutes of Health, Bethesda, MD, USA; Schneider et al., 2012).

### Scattering and absorption coefficients

We verified our estimates for light scattering from different floral layers in *O. glazioviana* with those obtained using an optical model. Our optical model relies on the Kubelka–Munk theory for media that scatter and absorb light (Kubelka and Munk, 1931) and considers flowers as a stack of layers (Stavenga and van der Kooi, 2016; Appendix S1). It enables us to model the scattering and absorption coefficients for petal layers with variable thickness and homogeneous pigmentation. We calculated the scattering and absorption coefficients for an intact petal, a petal without the adaxial epidermal layer and an isolated mesophyll.

## RESULTS

### The cuticle contributes little to the overall reflection

The very first layer that the incident light encounters is a thin waxy layer, the cuticle. The difference between the refractive index of the air and the cuticle determines the amount of light that is reflected. The refractive index of air is *n*_0_ = 1.00. Taking a refractive index of *n*_1_ = 1.46 for cuticular wax (Vogelmann, 1986, 1993; Bukhanov et al., 2020), Fresnel's equation for normally incident light *R* = [(*n*_1_ – *n*_0_)/(*n*_1_ + *n*_0_)]^2^ yields *R* = 0.03. In other words, under normally incident light, only 3% is reflected by the cuticle.

### Species varied markedly in internal flower structure, but their mesophyll similarly scattered light

The three studied species varied markedly variation in the thickness and internal structure of the different floral layers (Table 1). The petals of *T. majus* and *O. glazioviana* were of similar thickness, whereas the petals of *H*. ‘Hidcote’ were about four times thicker. The adaxial and the abaxial epidermis had a comparable thickness among the three species, whereas the mesophyll thickness varied greatly.

**Table 1:** Thickness of petal layers of *Tropaeolum majus*, *Hypericum* ‘Hidcote’ and *Oenothera glazioviana*. Intact: unmodified petal.

	Mean (±SD) thickness of layer (µm)			
Species	Intact	Adaxial epidermis	Mesophyll	Abaxial epidermis
*T. majus*	118 ± 15	53 ± 7	24 ± 13	40 ± 9
*H*. ‘Hidcote’	533 ± 30	35 ± 7	459 ± 27	39 ± 7
*O. glazioviana*	155 ± 10	24 ± 1	111 ± 12	19 ± 3

**Figure 2:**
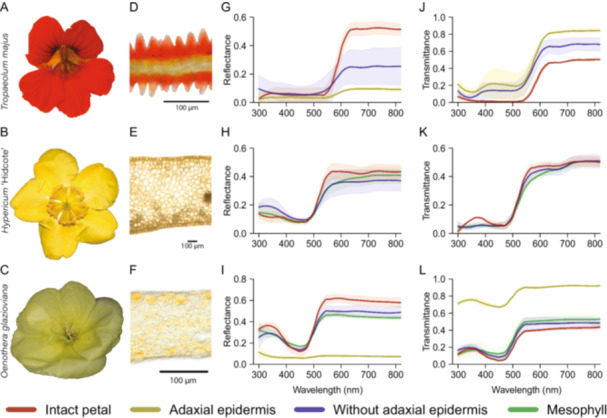
Top view of flowers (A–C), cross sections of fresh petals (D–F), spectral reflectance (G–I), and transmittance (J–L) of *Tropaeolum majus*, *Hypericum* ‘Hidcote’ and *Oenothera glazioviana*. Note that the range of the *y*‐axes varies.

Figure 2 shows the detached, whole flowers of the studied species (A–C), cross sections of fresh petals (D–F) and distribution of pigments, and the reflectance (G–I) and transmittance (J–L) spectra of the measured intact petal and its layers. As explained in the Materials and Methods (section Petal peeling, slide preparation, and spectrometry), we could not obtain all four layers for each species. To quantify light scattering by the floral layers, we focused on reflectance and transmittance at 800 nm, where absorption by pigments is negligible. Reflectance was highest in intact flowers and decreased when layers were removed. The opposite occurred for the transmittance (Appendix S2). In *O. glazioviana* and particularly in *T. majus*, the reflectance was markedly lower when the epidermal layers are removed (Figure 2G, I). For *H*. ‘Hidcote’, the reduction in reflectance when the epidermal layer was removed was small, indicating that most light is reflected by subepidermal tissue. Indeed, for *H*. ‘Hidcote’ the reflectance and transmittance (Figure 2H, K) of the intact petal and mesophyll were almost identical, suggesting that the contribution of the epidermal layer is negligible and that almost all reflection occurs in the mesophyll.

### Scattering varied among species and layers

The scattering coefficient is the scattering parameter divided by the thickness, so it serves as a measure of floral internal inhomogeneity. The scattering coefficients of the studied species at 800 nm are shown in Table 2. In the thick flowers of *H*. ‘Hidcote’, the scattering coefficients were low for all layers, indicating that the layers have a similarly low inhomogeneity. Therefore, the overall reasonably high reflectance of *H*. ‘Hidcote’ flowers (0.4 at long wavelengths, Figure 2H) is caused by the high thickness of the flower—especially that of the mesophyll—and not because of a high internal inhomogeneity. For intact petals, *T. majus* had the highest scattering coefficient (8.2 mm^−1^), followed by *O. glazioviana* with a scattering coefficient of 3.2 mm^−1^. The scattering coefficients for the isolated adaxial epidermis of these species are 1.8 mm^−1^ and 3.1 mm^−1^ (Table 2), due to the 2‐fold difference in epidermal layer thickness (40 µm for *T. majus*, 17 µm for *O. glazioviana*; Table 1). Another clear difference was in the shape and sizes of the epidermal cells, which were slightly convex and of variable sizes in *O. glazioviana* but cone‐shaped in *T. majus*. Although epidermal cell shape determines a flower's spatial reflection characteristics (Stavenga et al., 2020), our reflectance measurements were obtained with an integrating sphere that samples the entire hemispherical reflection, hence negating any variation in directionality. Our results thus suggest that the shape of epidermal cells does not crucially determine the amount of reflected light.

**Table 2:** Scattering coefficient (at 800 nm) for different petal layers from *Tropaeolum majus*, *Hypericum* ‘Hidcote’ and *Oenothera glazioviana*. Exposed mesophyll: only the adaxial epidermis removed to revealing the mesophyll layer; mesophyll: adaxial and abaxial epidermis removed.

	Mean (±SD) scattering coefficient (mm^−1^)			
Species	Intact petal	Exposed mesophyll	Mesophyll	Adaxial epidermis
*T. majus*	8.2 ± 0.2	3.9 ± 1.8	—	1.8 ± 0.7
*H*. ‘Hidcote’	1.4 ± 0.2	1.2 ± 0.3	1.5 ± 0.3	—
*O. glazioviana*	3.2 ± 1.1	3.3 ± 0.1	1.7 ± 0.3	3.1 ± 0.1

### Absorbance of floral layers

**Figure 3:**
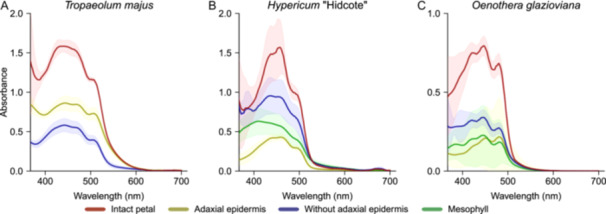
Absorbance spectra of petal layers in (A) *Tropaeolum majus*, (B) *Hypericum* ‘Hidcote’, and (C) *Oenothera glazioviana*. Note that the range of the *y*‐axes varies.

To evaluate the effect of pigmentation in different floral layers, we determined the relative absorbance of each layer (Appendix S3). Absorbance is dimensionless and is expressed at a logarithmic scale, indicating the amount of incident light absorbed by a given sample. The absorbance peak wavelength is located at 450 nm for all species and for intact petals (Figure 3), which is characteristic for carotenoids (Grotewold, 2006; Lee, 2007; Narbona et al., 2021). The peak absorbance decreased when a floral layer was removed. Removal of the adaxial epidermis resulted in a significant reduction in absorbance, suggesting that pigments are distributed asymmetrically among floral layers. Removal of the adaxial epidermal layer reduced the absorbance from 1.6 to 0.6 in *T. majus* (Figure 3A), from 1.4 to 0.9 in *H*. ‘Hidcote’ (Figure 3B) and from 0.8 to 0.3 in *O. glazioviana* (Figure 3C), indicating that the epidermal layers contained a considerable amount of pigment. In *T. majus*, the absorbance of the epidermis was higher than that of the exposed mesophyll layer (Figure 3A), whereas for *H*. ‘Hidcote’ and *O. glazioviana* absorbance of the exposed mesophyll was higher than for the isolated mesophyll and isolated epidermis (Figure 3B, C). Another clear difference was the variation in standard deviations (Figure 3), likely owing to variability in thickness of single layers, which could be caused by co‐removal of cells from an underlying layer when the adjacent layer was removed.

### Scattering and absorption coefficients

**Figure 4:**
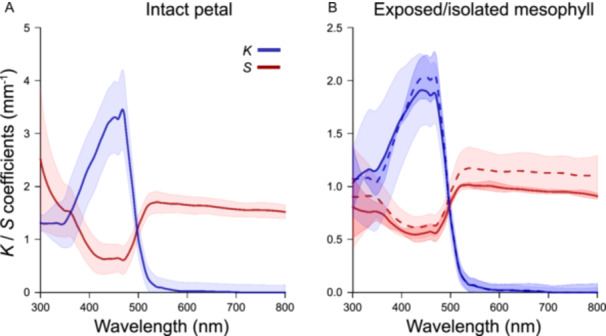
Optical model of different petal layers in *Oenothera glazioviana*. (A) Intact petal and (B) exposed mesophyll (dashed lines) versus isolated mesophyll. *K*, absorption parameter; *S*, scattering parameter.

Modelling showed that wavelength absorption by pigments in *O. glazioviana* significantly reduced light scattering from a layer, a trend that was present in all examined layers (Figure 4). For intact petals, the absorption coefficient at 450 nm was 3.5 mm^–1^, whereas the scattering coefficient was 0.5 mm^–1^ (Figure 4A). At 800 nm, where there is no absorption by pigments, the values of scattering increased to 1.5 mm^–1^. In the experimental spectra and the model, the values for absorption and scattering decreased as the thickness of the layer decreased.

The model showed that the abaxial and the adaxial epidermis had a similarly small role in light absorption/reflection. The scattering/absorbance of the “exposed” mesophyll (i.e., the mesophyll plus the abaxial epidermis) was very similar to that of the isolated mesophyll with both epidermal layers removed. Therefore, both epidermal layers contributed little to the scattering. This highlights the predominant role of floral mesophyll in absorption and scattering of light.

## DISCUSSION

Floral structures play a crucial role for visual signalling, but how different layers and structures contribute to scattering of light is largely unknown. Using spectroscopy and optical modelling, we estimated the layers' relative contributions to the overall visual signal for three species with similar pigmentation (Figure 3). Even though our taxon sampling was not exhaustive and included a *Hypericum* hybrid that does not occur in nature, our observations revealed several insights into the mechanistic underpinnings of light scattering by different tissue layers in petals.

**Figure 5:**
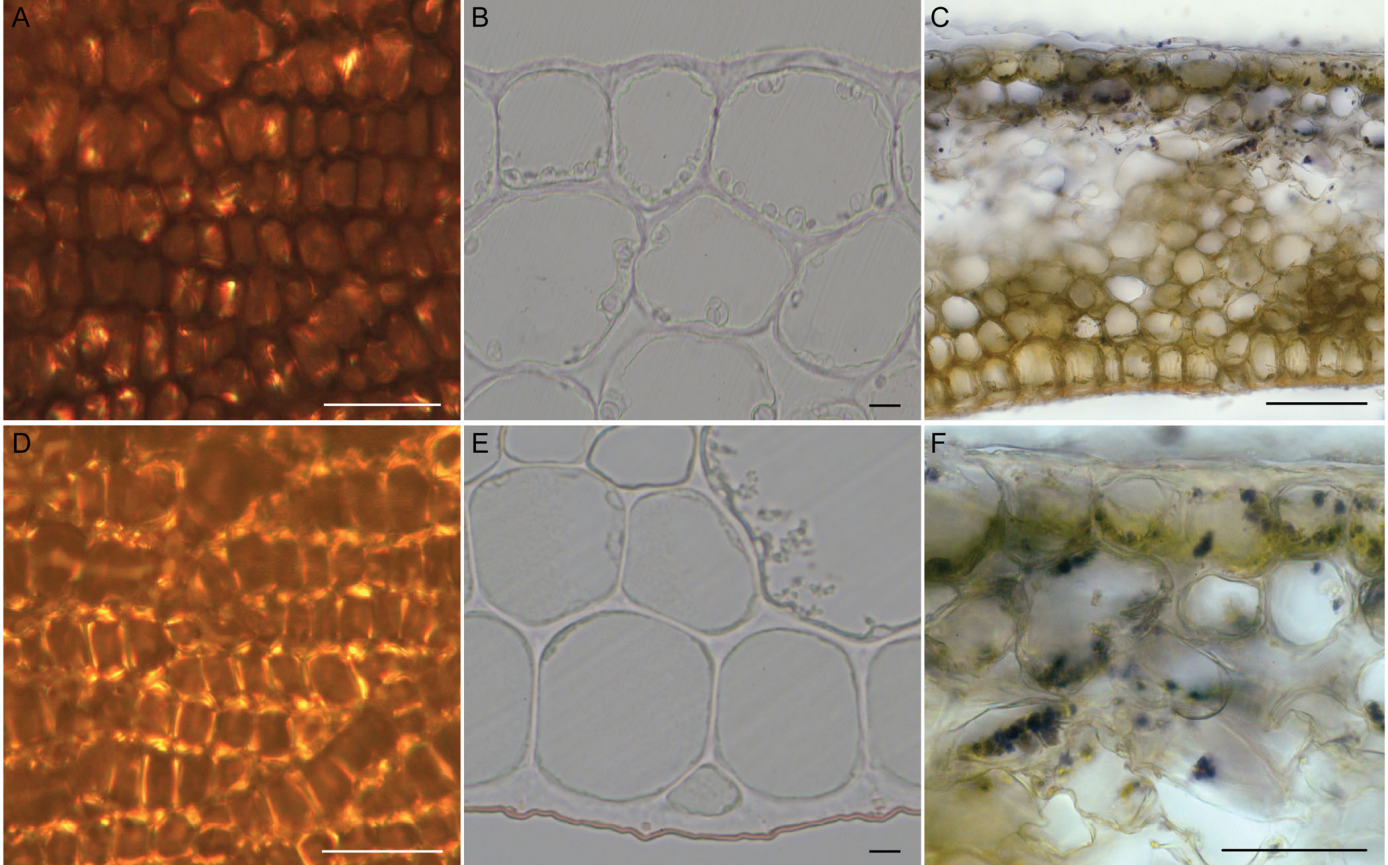
Examples of specific scattering floral structures. (A, C) Epidermal cells in *Oenothera glazioviana*. The curvy, serpentine epidermal cell walls appear dark in transmission (A) and bright in reflection (C), indicating that the cell walls are strongly scattering. (B, E) Epidermal cells in *Hypericum* ‘Hidcote’ stained with Sudan red to highlight the cuticle. The adaxial epidermal cells (B) show (almost) no cuticle, but the cuticle is prominently visible on the abaxial side (E). (C, F) Starch granules (the black dots) appear across the adaxial side of the mesophyll in *H*. ‘Hidcote’. Scale bars: 50 µm (A, D, F), 10 µm (B, E), 100 µm (C).

The cuticle is the very first layer that incident light encounters when it hits a flower. Cuticles are ubiquitous among flowering plants (Lee, 2007), though most research on the cuticle has been on leaves (Vogelmann, 1993; Barthlott and Neinhuis, 1997; Neinhuis and Barthlott, 1997; González Moreno et al., 2022). Floral cuticle presumably plays a role in hydrophobicity (e.g. Taneda et al., 2015) and protection from UV light (e.g. González Moreno et al., 2022). Two recent comparative studies showed that the flower cuticle is significantly less waxy and more permeable than the leaf cuticle of the same species (Roddy et al., 2023; Tunstad et al., 2024). In line with these findings is our observation that the cuticle of *H*. ‘Hidcote’ is hardly noticeable on the adaxial epidermis but is prominently visible on the abaxial epidermis (compare the red‐stained cuticle in Figure 5B, E). The contribution of the cuticle to the total floral reflection is minor. The cuticle reflectance is about 0.03—a value that is independent of the cuticle's thickness because light is reflected at the boundary of two media, regardless of their thickness. Leaf cuticle has a similarly low reflectance (Lee, 2007). Finally, light that is reflected by the cuticle will not be modulated by pigments, meaning that any surface reflections are spectrally featureless. Such unmodulated (white with ultraviolet) reflections are poorly visible to insects (Kevan et al., 1996), so they should be selected against in the context of visual signalling.

The adaxial epidermal layer played a more prominent optical role. The contribution of the epidermal layer to the total reflectance was around 0.08 to 0.1 (Figure 2). The epidermal layer is only one cell layer thick (~20–50 µm), so its scattering coefficient was comparatively high. Indeed, the scattering coefficient of the adaxial epidermal layer was higher than that of the mesophyll. Serpentine cell walls such as those in *O. glazioviana* (Figure 5A, D) and poppy flowers (van der Kooi and Stavenga, 2019) strongly reflect light because the air pockets that adhere to the curvy walls enhance local inhomogeneity. The epidermal layer is also important for the floral visual signal because anthocyanin pigments are concentrated in the epidermal vacuole (Grotewold, 2006; Lee, 2007) and because epidermal cell shape determines the degree of surface gloss (Stavenga et al., 2020).

Most light is reflected by the mesophyll. The mesophyll of *O. glazioviana* reflects much more light than the adjacent epidermal layer does, because the mesophyll is four times thicker than the epidermis. A similar case occurs in the thin flowers of *T. majus*, although the different layers were difficult to separate and the reflectance values were more variable. The high variability for the exposed mesophyll (i.e., the petal without its adaxial epidermis, Figure 2G) is because removal of the epidermis disturbs the structure of the thin mesophyll underneath. Despite the high variability, in *T. majus*, the exposed mesophyll scattered more light than the isolated epidermis, showing that even very thin mesophyll tissue (~20 µm) can be the main source of light scattering in the petal. The contribution of the abaxial layer to the total reflection was minor (Figure 4B) because most light is scattered before it reaches the abaxial epidermis.

How similar or different are the mesophyll of leaves and petals, and is there evidence for tuning of petal mesophyll to create visual signals that are conspicuous to pollinators? The optical functions of leaves and flowers are opposite: optimizing light capture for photosynthesis versus optimizing visual contrast through light reflection. Leaves and flowers are both characterized by a porous mesophyll that contains air gaps and other structures that scatter light. In leaf mesophyll, internal scattering may increase the possibility for light absorption by chlorophyll in the palisade cells, whereas backscattering in perianths enhances the perceived brightness of the visual signal. Localization of floral pigments in the cell layer on the side that is viewed (e.g., in adaxial epidermal cells) can further enhance the wavelength‐selective modulation of the reflected light (van der Kooi et al., 2016; Fan et al., 2024). The literature on floral mesophyll structure and inhomogeneity is limited, but a recent study by Schreel et al. (2024) hints at possible tuning of mesophyll porosity in leaves and flowers. Investigating the differences between the mesophyll of leaves and perianths of six species, Schreel et al. (2024) found that perianth mesophyll is significantly more porous than leaf mesophyll. An increase in porosity caused by air pockets is one of the most effective ways to enhance scattering because of the high differences in the refractive index of air and that of plant material. Too many air pockets will, however, create an essentially “hollow” flower, which undermines mechanical strength and scattering. In leaves, mesophyll density and mesophyll cell wall properties are also affected by the degree of shading (Luo et al., 2023). Starch granules, such as those observed in *H*. ‘Hidcote’ (Figure 5C, F), also strongly scatter incident light, but it is presently unclear how common starch grains are among flowers. Combining the structural differences between leaf and perianth mesophyll (Schreel et al., 2024) and our observation of the central role for mesophyll for floral light scattering, we conclude that mesophyll thickness and porosity are key to floral visual signalling.

Our findings on the importance of mesophyll for floral visual signalling open avenues for future research on floral signalling and development. Research on floral scattering structures has primarily focused on floral cuticle structure and epidermal cell shape (reviewed by Lee, 2007 and van der Kooi et al., 2019); however, it seems intuitive that the mesophyll is more evolutionarily labile than cuticle and epidermal cell properties. The cuticle and epidermal cells constitute the flower's outer boundary to the environment, so are subject to additional constraints, such as biomechanic and dehydration properties. Given its prominent role for floral signalling, the mesophyll may be a target for natural selection. For example, when pollinators or the abiotic environment select for enhanced floral brightness, flowers may evolve to have thicker and/or more porous mesophylls. Floral achromatic (“brightness”) contrast is important for detection of flowers by insects, such as bees (Giurfa et al., 1996; Dyer et al., 2008; Meena et al., 2021), flies (An et al., 2018), hawkmoths (van der Kooi and Kelber, 2022), and butterflies (Koshitaka et al., 2011; Kinoshita et al., 2012). A moderate backscattering is sufficient in most cases because, for diurnal pollinators, modulation of the reflected light by floral pigments (i.e., color contrast) is more important than high “brightness” (Daumer, 1956; Chittka et al., 1992; Kevan et al., 1996; Spaethe et al., 2001; Goyret and Kelber, 2012; Papiorek et al., 2013; van der Kooi et al., 2019). In dim light, high floral scattering of light may provide an adaptive benefit because brighter signals become more conspicuous (Borges et al., 2016; van der Kooi and Kelber, 2022). An example of a strongly scattering, nocturnal flower may be *Oenothera glazioviana*, a species predominantly pollinated by hawkmoths (Kawaano et al., 1995).

## CONCLUSIONS

Owing to its crucial role in light scattering and contribution to the visual signal, the mesophyll is a hitherto underappreciated component of the optical toolkit of flowers. Changes in thickness, inhomogeneity, and porosity of mesophyll will modulate the scattering of light by flowers. Floral mesophyll structure can be tuned to scatter more light through pigmented (epidermal) layers and thus enhance the efficiency of floral pigments. What the optimal ratio of air pocket to cellular structure is for visual signalling and/or abiotic roles of (internal) scattering requires further study. In a broader evolutionary context, the study of scattering of light in other signalling organs, such as the vividly colored bracts in species such as *Anthurium*, *Musa*, or the cyathophylls in *Euphorbia* will reveal how angiosperms that lack showy flowers can produce a conspicuous visual signal.

## AUTHOR CONTRIBUTIONS

L.D.P. and T.V. did all spectral measurements and analyses and drafted the manuscript under the guidance of C.J.v.d.K. and D.G.S. M.K. produced the cross sections. K.J.T. provided input on the analyses. All authors contributed to writing or revising the manuscript.

## Supporting information


**Appendix S1.** Cross sections of fixed and fresh petals and calculations for scattering and absorption coefficients.

**Appendix S2.** Reflectance and transmittance spectra measured using an integrating sphere.

**Appendix S3.** Absorbance spectra measured with the microspectrophotometer.

## Data Availability

Reflectance and transmittance spectra are included in Appendix S2 and absorbance spectra are in Appendix S3.
