# Supplementary material for: Stacked scattering: The key to bright flowers lies in the mesophyll
Source: Am J Bot. 2025 Sep 25;113(1):e70104. doi: 10.1002/ajb2.70104 (PMC12816439; doi:10.1002/ajb2.70104)
Supplement: Supplementary file 1 — Appendix S1. Cross sections of fixed and fresh petals and calculations for scattering and absorption coefficients. [file AJB2-113-e70104-s002.docx]

**Appendix S1.** Cross sections of fixed and fresh petals and scattering and calculations for absorption coefficients.

The images below are cross sections of fixed or fresh petals. Top row: *Hypericum* ‘Hidcote’, fixed, stained with toludine blue. Middle row: *Oenothera glazioviana,* left and middle, fixed, stained with toluidine blue; right, fresh. Bottom row: *Tropaeolum majus*. Images, taken with an inverted Nikon Diaphot 300 microscope and D3200 digital camera, were used to calculate the thicknesses in Table 1. Scale bars = 100 µm.


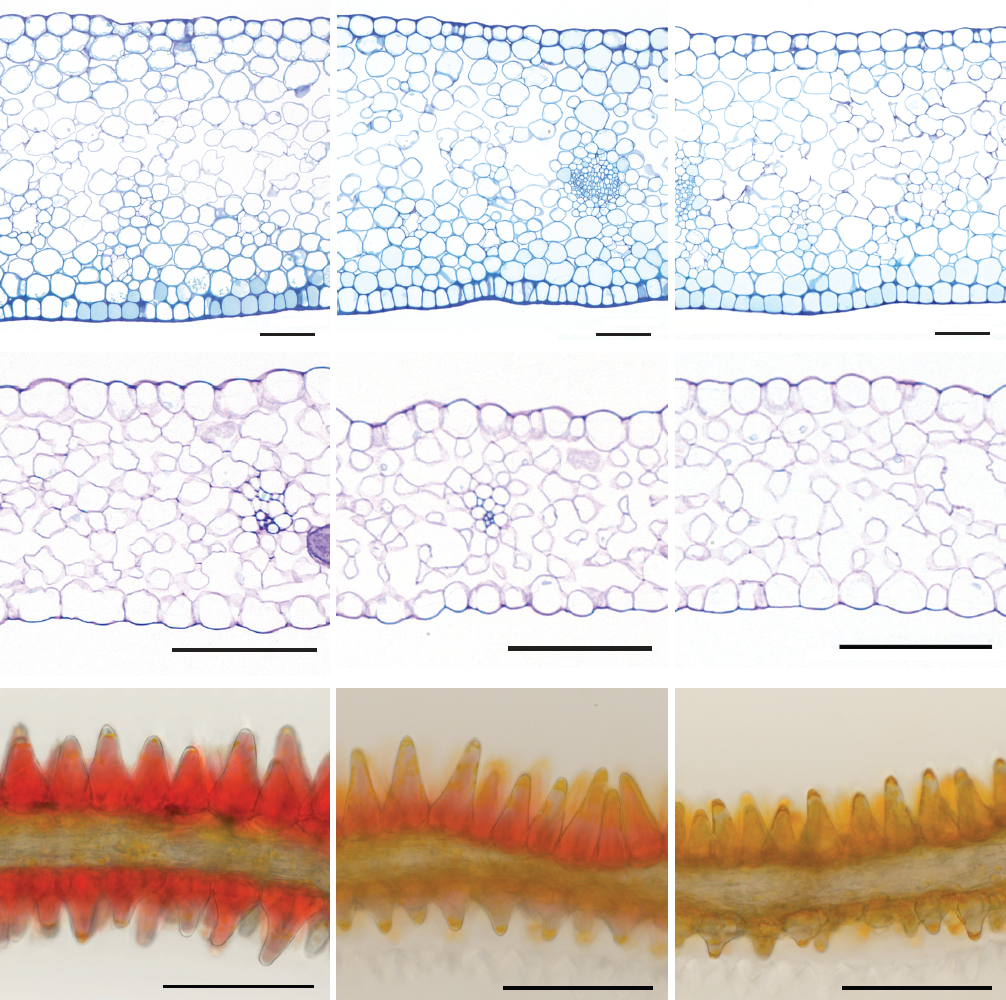


**Scattering and absorption coefficient calculations**

The scattering coefficient *S* is a measure of the light scattered per unit thickness (*d*) and calculated as *S = S*/d*. The scattering parameter *S** is calculated as *S** *= R/*(1 – *R*) at 800 nm only because pigments do not absorb this wavelength. In our optical model, we considered all light wavelengths, so the scattering parameter (*S**) was also calculated as $S^{*}=ln \{[1-(a-b)R]/T\}$ $\div b$, where $=(1+R^{2}-T^{2}/2R$ and *b* = $\sqrt{a^{2}-1}$.

Finally, we calculated the absorption coefficient, *K* = *K*/d*, which is a measure of absorption per unit thickness (*d*), defined as *K** = (*a* – 1)*S**.
